# SynSigGAN: Generative Adversarial Networks for Synthetic Biomedical Signal Generation

**DOI:** 10.3390/biology9120441

**Published:** 2020-12-03

**Authors:** Debapriya Hazra, Yung-Cheol Byun

**Affiliations:** Department of Computer Engineering, Jeju National University, Jeju 63243, Korea; debapriyah@jejunu.ac.kr

**Keywords:** biomedical signals, generative adversarial networks, synthetic data, health care, EEG, ECG, EMG, PPG

## Abstract

**Simple Summary:**

This paper proposes a novel generative adversarial networks model, SynSigGAN, to generate any kind of synthetic biomedical signals. The generation of synthetic signals eliminates confidentiality concerns and accessibility problem of medical data. Synthetic data can be utilized for training medical students and machine learning models for the advancement and automation of healthcare systems. Our proposed model performs significantly better than existing models with a high correlation coefficient that measures the generated synthetic signals’ similarity with the original signals.

**Abstract:**

Automating medical diagnosis and training medical students with real-life situations requires the accumulation of large dataset variants covering all aspects of a patient’s condition. For preventing the misuse of patient’s private information, datasets are not always publicly available. There is a need to generate synthetic data that can be trained for the advancement of public healthcare without intruding on patient’s confidentiality. Currently, rules for generating synthetic data are predefined and they require expert intervention, which limits the types and amount of synthetic data. In this paper, we propose a novel generative adversarial networks (GAN) model, named SynSigGAN, for automating the generation of any kind of synthetic biomedical signals. We have used bidirectional grid long short-term memory for the generator network and convolutional neural network for the discriminator network of the GAN model. Our model can be applied in order to create new biomedical synthetic signals while using a small size of the original signal dataset. We have experimented with our model for generating synthetic signals for four kinds of biomedical signals (electrocardiogram (ECG), electroencephalogram (EEG), electromyography (EMG), photoplethysmography (PPG)). The performance of our model is superior wheen compared to other traditional models and GAN models, as depicted by the evaluation metric. Synthetic biomedical signals generated by our approach have been tested while using other models that could classify each signal significantly with high accuracy.

## 1. Introduction

Deep learning has spurred significant advances in the healthcare industry. Its technological developments have not only provided solutions to intricate problems, but also reduced costs and enhanced efficiency in the medical domain. It was predicted by the International Data Corporation (IDC) in the year 2019 that the worldwide market for artificial intelligence (AI), deep learning, and machine learning would reach 97.9 billion US dollar by 2023 with a compound annual growth rate of 28.4% [1]. Currently, deep learning algorithms utilize data provided by the electronic health records in order to predict risk factors, detect health patterns, and correctly diagnose diseases. Deep learning is not only used prediction, but also for the analyzing, tracking, and detection of health conditions or particular diseases. Nvidia published a study where advanced algorithms were able to reduce medical misdiagnosis in cancer by 85% [2]. Researchers around the world collaborated in order to apply deep learning for predicting heart failures from electronic health records up to nine months earlier than doctors manually can. Renowned companies, like Google, created convolutional neural network models to build LYmph Node Assistant (LYNA), which achieved 99% accuracy as compared to doctors who achieved 38% accuracy in detecting metastasized breast cancer. Over 415 million people in the developing countries suffer from diabetic retinopathy, which subsequently leads to blindness. Machine learning models use retinal images to detect early symptoms of diabetic retinopathy and they eliminate the potential risk of blindness in patients. Not only this, but reinforcement learning models, are currently used to track biomarkers and plan daily drug dosage courses for continuous treatment of Human Immunodeficiency Virus (HIV) [2].

Despite many advantages of AI and deep learning models, one of the risks involved is the vulnerability of private data that are stored in electronic health records. Patient’s privacy being a major concern of the hospitals, acquiring large datasets in order to train any model becomes very difficult. The impact of deep learning, AI, and machine learning in healthcare research has been relatively slower and complicated due to the lack of availability of medical data and low accessibility for potential researchers. Even if the data are accessible to the researcher, the legal requirements to acquire data by ensuring proper and protected usage of the data takes a considerable amount of time and, thereby, delays the development of advanced technology that could benefit the healthcare sector. To make medical data available, many owners anonymize data by detaching recognizable features, adding noise and grouping individuals or variables into broader categories [3]. However, the re-identification of those anonymized data in the large dataset becomes extremely difficult, time consuming, and, therefore, not approachable. Generating synthetic medical data is a solution to this problem and it is considered to be valuable when there is insufficient original data or original data are expensive. Synthetic data can be used exclusively or with the original data for training purposes, education, machine learning model development and software testing. There are many methods to generate synthetic data, but one of the most successful frameworks for generating synthetic medical data is generative adversarial networks (GAN). Understanding physiological signals and generating synthetic biomedical signals is a complicated and much required task. Each biomedical signal has its own morphological characteristics, depending on the source of organ, independent traits, noise, electrode placement, and pathological events. External devices also corrupt the quality of biomedical signals, with excessive noise making them unreadable.

In this paper, we propose a novel generative adversarial network, named as SynSigGAN, for generating synthetic biomedical signals using a small dataset of real signals. The main contribution of this paper is summarized, as below:

1. We propose a preprocessing stage to refine the biomedical signals while using a combination of discrete wavelet transform (DWT), thresholding, and Inverse discrete wavelet transform (IDWT). This model can be modified according to the signal requirement and it can be used for denoising and refining any kind of signals.
2. Our proposed generative adversarial networks model can be applied in order to generate any kind of biomedical signals.
3. We have an evaluation stage to ensure the authenticity and similarity of synthetic data as compared to the original data.
4. As the data enhance, the approach can be reused in order to generate more synthetic data.

After the generation of the synthetic data, we evaluate the Pearson correlation coefficient to verify the quality of synthetic data when compared to the original data. We also calculate the Root Mean Square Error (RMSE), Percent Root Mean Square Difference (PRD), Mean Absolute Error (MAE), and Frechet Distance (FD) for statistical analysis. The results show that our model outperforms existing models and generates synthetic biomedical signals that can be practically used for the advancement of healthcare industry.

The rest of this paper includes related works in Section 2, an explanation of datasets used for our work in Section 3, the proposed methodology for generating synthetic data in Section 4, evaluation and results in Section 5, and Section 6 concludes our proposed work.

## 2. Related Works

There is a large variation of machine learning and deep learning models available in the literature for experimenting with biomedical signals. Additionally, synthesized signals have been assessed for signal denoising, event detection, reconstructing illegible signals, classification, and generation of biomedical signals.

Patrick E. McSharry et al. proposed a dynamic model that was built on three coupled ordinary differential equations for generating synthetic electrocardiogram signals [4]. The generation of realistic synthetic electronic health records without the use of real electronic health records was proposed in [5]. They used the CareMap with health incidents statistics to achieve generating synthetic electronic health records. A. D. Moore synthesized Electromyography signals using the integration of diphasic waves and multiplication of sinus waves along with autoregressive models mixed with gaussian noise [6]. I.S.N. Murthy et al. generated synthetic data for electrocardiogram signals while using discrete cosine transform [7]. Radford et al. presented unsupervised learning and generated synthetic images while using deep convolutional generative adversarial networks (DCGAN) [8]. Fei Zhu et al. proposed a bidirectional long short-term memory-convolutional neural network GAN for electrocardiogram generation [9]. Fei Zhu et al. claimed that their model could generate ECG data with relatively high morphological similarity when compared to real ECG data.

Generative adversarial networks are now an emerging framework to deal with images and time series data and also for the creation of synthetic data. Stephanie Hyland et al. used recurrent conditional GANs (RCGAN) for the synthetic generation of real-valued medical time series data [10]. They evaluated their model and concluded that RCGAN can generate time series data that are useful for supervised training but with minor degradation in performance on real test data. Annie Marie Delaney et al. proposed generative adversarial networks for realistic synthetic ECG signal generation [11]. They tested their generator with two models, namely LSTM and Bidirectional LSTM, and for the discriminator they compared LSTM and CNN. The generator architecture with LSTM consisted of two layers of LSTM, followed by a fully connected layer that generated synthetic ECG data. They used 50 hidden layers in both of the LSTM layers. For the generator architecture with BiLSTM, they used two BiLSTM layers, along with a fully connected layer and 50 hidden layers. For the discriminator built with LSTM, the authors used two LSTM layers with 50 hidden layers, a minibatch discriminator layer, and a fully connected layer with a sigmoid activation function for classification. The CNN discriminator was built using a convolutional pooling layer with a ReLu activation function, followed by a minibatch discriminator and a fully connected layer for classification. Their model depicted that GAN with two bidirectional LSTM in the generator and convolutional pooling layer in the discriminator performed the best and generated high-quality ECG data.

In [12], data scientists and researchers concluded that, statistically, there is no significant difference between the original and synthetic data. Models have been trained and evaluated for classification, prediction and detection of diseases with only synthetic data and also with original data solely. In cases where there is a combination of synthetic data and original data or only synthetic data, the acquired results have been more accurate. Many physicians have confirmed that there are many cases where synthetic data met specific rare conditions that original data could not. These synthetic data can be integrated with wearable smart healthcare devices and trained in order to build models for emergency alert generation [13]. COCOA is framework that was presented by Vanessa Ayala-Rivera et al. for generating realistic synthetic microdata that can preserve the functional dependencies of data by allowing multi-attribute relationships [14].

Medical generative adversarial networks that are known as medGAN are a famous approach for creating realistic synthetic patient records. It generates high-dimensional discrete values by combining autoencoders and GAN architecture [15]. Authors have used autoencoders in medGAN to learn salient features by relating samples to lower dimensional space and, afterwards, projecting samples to the original space. Instead of directly generating patient records, GAN has been provided here with pre-trained autoencoders to generate distributed patient data. Convolutional generative adversarial networks have also been used for obtaining synthetic healthcare data [16]. The authors have utilized one-dimensional (1-D) convolutional neural networks to apprehend the correlation between consecutive diagnosis data. Subsequently, they used convolutional autoencoders to relate discrete-continuous values. Finally, fidelity and privacy risk was measured in order to conclude the proposed work. Private Aggregation of Teacher Ensembles (PATE) is another GAN model for generating synthetic data that ensures differential privacy of the generator which is significantly important for biomedical data [17]. In PATE-GAN, the generator resembles the generator of a standard GAN model, but the discriminator is built with PATE mechanism, so there are k-teacher discriminators and the training is asymmetrical. Synthesizing normal heart sounds while using GAN were proposed by Pedro Narvaez et al. [18]. They used their data as a test dataset to evaluate heart sound classification models. Brain signals or EEG data have also been synthetically generated using GAN models by many researchers. One of the models is named as neural-based generative models for SSVEP classification in EEG data [19]. Authors used deep convolutional generative adversarial networks and variational autoencoders for this purpose. A recent work also produced efficient results for generating synthetic biomedical singals while using bidirectional recurrent neural network [20].

Therefore, as the related studies depict, a lot of work has been done for the generation of synthetic medical data, including signals. However, less work has been done in order to create generalized model for generating synthetic data for all kinds of biomedical signals. In the section below, we describe the data that were used for signals and propose a methodology for generating synthetic signals.

## 3. Data

We have trained and evaluated our proposed model both qualitatively and quantitatively with four biomedical signal databases [21]. The biomedical signals considered in our work are electrocardiogram (ECG), electroencephalogram (EEG), electromyography (EMG), and photoplethysmography (PPG). We have acquired the ECG data from MIT-BIH Arrhythmia Database [22]; EEG data from Siena Scalp EEG Database [23,24]; EMG data from Sleep-EDF Database [25]; and, PPG data from the BIDMC PPG and respiration Dataset [26]. Description of the datasets are mentioned in the following subsections.

### 3.1. MIT-BIH Arrhythmia Database

The MIT-BIH Arrhythmia Database is a collection of digitized and annotated long-term ECG recordings from Boston’s Beth Israel Hospital for arrhythmia analysis. The ECG recording from 47 subjects and a total of 201 records were obtained from 48 half-hour extracts of two-channels. Twenty-three recordings were randomly chosen from 4000 Holter tapes and the rest 25 were a collection of clinically important arrhythmia’s that are generally uncommon in small samples. Among the 47 patients, 25 were men aged 32 to 89 years, and 22 women from 23 to 89 years. Mostly, one of the two channels were modified limb lead II (MLII) and the other channel was among V1, V2, V4, or V5, depending upon the patients. The sampling frequency was 360 Hz and the signal duration was 30 min. The 17 types of ECG signals that are present in the MIT-BIH Arrhythmia Database are as follows:

- Normal Beat (NB)
- Rhythm Change (RC)
- Right Bundle Branch Block Beat (RBBB)
- Left Bundle Branch Block Beat (LBBB)
- Ventricular Escape Beat (VEB)
- Atrial Premature Beat (APB)
- Premature Ventricular Contraction (PVC)
- Nodal (Junctional) Escape Beat (NEB)
- Aberrated Atrial Premature Beat (AAPB)
- Fusion of Ventricular and Normal Beat (FVNB)
- Fusion of Paced and Normal Beat (FPNB)
- Ventricular Flutter Wave (VFW)
- Comment Annotations (CA)
- Paced Beat (PB)
- Non Conducted P Wave (Blocked APC) (NCPW)
- Change in Signal Quality (CSQ)
- Unclassifiable Beat (UB)

### 3.2. Siena Scalp EEG Database

The Siena Scalp EEG Database was obtained from the Unit of Neurology and Neurophysiology at University of Siena. The EEG recordings are of 14 patients, which includes nine males aged 25–71 and five females of age 20–58. The sampling rate was 512 Hz for the diagnosis of epilepsy and seizure classification. The database has recordings in European Data Format (EDF). Each patient has between one and five data files of maximum 2.11 GB size and also a text file containing seizure and data information. The dataset has an excel file containing gender, age, seizure classification, number of EEG channels, total number of seizures, and recording time in minutes. Seizures are classified as IAS, WIAS, and FBTC. The dataset contains a total of 47 seizures and recording of 128 h.

### 3.3. Sleep-EDF Database

Polysomnographic sleep recordings of 197 full-nights containing chin EMG recordings are obtained from the Sleep-EDF Database. The database has two types of files, namely Sleep Cassette (SC) study and Sleep Telemetry (ST) study files. Each file contains *PSG.edf, which are polysomnographic sleep recording files, and *Hypnogram.edf, which are the sleep pattern annotation file that corresponds to the PSG files. The signals were sampled at 100 Hz and event marker at 1 Hz. There are 153 SC files that study the effects of age on sleep for age group of 25 to 101 and 44 ST files from 22 males and females.

### 3.4. BIDMC PPG and Respiration Dataset

The signals in the BIDMC PPG and respiration dataset are extracted from the MIMIC II matched waveform database, with breath annotations being done manually by two annotators using the impedance respiratory signal. The dataset is acquired from Beth Israel Deaconess Medical Centre (Boston, MA, USA). There are 53 recordings, each of which is of 8 min duration. Each recording contains:

- Physiological signals sampled at 125 Hz.
- Parameters such as respiratory rate, heart rate and blood oxygen saturation level which are sampled at 1 Hz.
- Fixed parameters such as age and gender.

## 4. Proposed Methodology for Generating Synthetic Biomedical Signals

**Figure 1:**
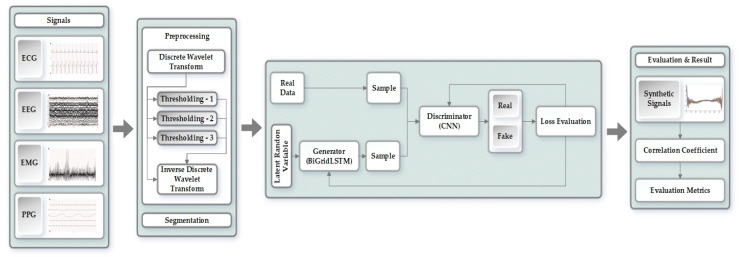
Overview of the proposed approach.

The proposed methodology for generating synthetic biomedical signals has been divided into different stages as shown in Figure 1. We have processed and evaluated each of the four signals (ECG, EEG, EMG, and PPG) independently through the proposed approach. The original signals proceed through the preprocessing stage, eliminating noise and refining the signals while using discrete wavelet transform (DWT), thresholding, and inverse discrete wavelet transform (IDWT). After preprocessing, the signals are then forwarded to the segmentation stage that uses the Z-score to solve amplitude scaling problem and eliminate offset. Next is the generative adversarial networks model, which takes in the segmented signals and generates synthetic biomedical signals using bidirectional grid long short-term memory for generator network and convolutional neural network for the discriminator. In the last stage, we statistically evaluate our model to measure and prove the quality of the synthetically generated signals when compared to the original signals.

### 4.1. Preprocessing of the Original Signals

In the preprocessing stage, we have implemented a wavelet denoising mechanism for biomedical signals [27]. We have used wavelet transformation, since it produced a superior denoising performance for signals due to its multiresolution and windowing characteristics as compared to other mechanisms [28]. The original signals were processed through four Daubechies filters, namely, G(z) and H(z), which are basically four tap high-pass and low-pass filters, which can be represented as in (1):

(1) $$\begin{array}{c} G\left(z\right)=G_{0}z+G_{1}z^{-1}+G_{2}z^{-2}+G_{3}z^{-3}\\ H\left(z\right)=H_{0}z+H_{1}z^{-1}+H_{2}z^{-2}+H_{3}z^{-3} \end{array}$$

**Figure 2:**
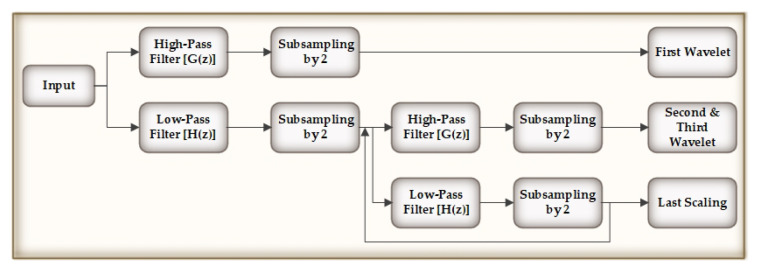
Discrete Wavelet Transform (DWT).

After this, we compute the detailed wavelet coefficients and approximate coefficients from the high and low-pass filters. The output of the low-pass filter is then subsampled by 2 and again processed through a high and low-pass filters with half cut-off frequency from the previous time, as shown in Figure 2. The frequency resolution is doubled, since the outputs are of half the frequency of the input.

In order to preserve sharp features of the biomedical signals, we incorporated thresholding that was applied to the wavelet coefficients generated by DWT. For each DWT level, we assigned an adaptive thresholding process on the wavelet coefficients that were created at that level. The adaptive threshold applied to i-th DWT level can be defined as (2):

(2) $$\begin{array}{c} AT_{i}\left(j\right)=\frac{1}{32}\sum_{n=32(j-1)}^{32j-1}\left|x_{1}\left(n\right)\right|\times 2^{i} \end{array}$$

where, $AT_{i}\left(j\right)$ is the j-th threshold value for performing adaptive thresholding in the i-th level of DWT and $x_{1}\left(n\right)$ is the wavelet coefficient in the first DWT level. 32 is an observed choice made for the moving window length. As the DWT level increases by one, the threshold value also rises by a factor of 2, as we can see from the following Equation (3). During thresholding, the average of the absolute values of the consecutive wavelet coefficients are computed and then the data sequences obtained from the DWT are fed to the IDWT for further processing.

(3) $$\begin{array}{c} AT_{i}\left(1\right)=\frac{1}{32}\sum_{n=0}^{31}\left|x_{1}\left(n\right)\right|\times 2^{i}\\ AT_{i}\left(2\right)=\frac{1}{32}\sum_{n=32}^{63}\left|x_{1}\left(n\right)\right|\times 2^{i}\\ AT_{i}\left(3\right)=\frac{1}{32}\sum_{n=64}^{95}\left|x_{1}\left(n\right)\right|\times 2^{i} \end{array}$$

**Figure 3:**
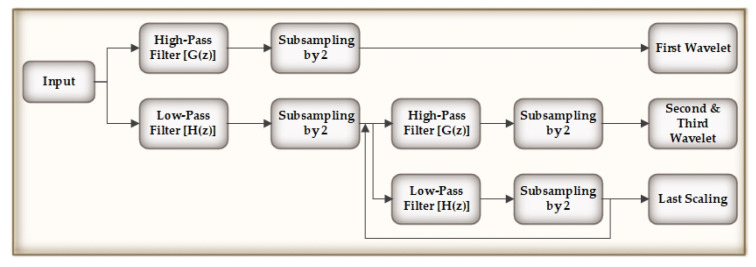
Inverese Discrete Wavelet Transform (IDWT).

In the last phase, the IDWT generates the denoised signals by determining the inverse DWT of the wavelet coefficients that has been thresholded. Figure 3 explains the IDWT flow. In Table 1, we show the performance comparison of other denoising techniques with our used methodology (wavelet transformation) through signal-to-noise ration (SNR), root mean square error (RMSE), and percentage root-mean-square difference (PRD).

### 4.2. Segmentation

In the segmentation stage, we cluster the signals according to the annotations that are mentioned in each of the datasets and then segment the signals. The later step is to concatenate the signals of similar type [29]. Another approach that was taken for segmenting ECG signals was to extract the median of R-R time intervals and consider it as the nominal heartbeat period (T). We have followed the work from [30] for ECG signal segmentation. We need the same class of signals with similar sizes to forward the signals to the adversarial networks.

Let us consider a recording that has two different types of ECG signals: normal and Ventricular Escape Beat (VEB). This can be represented while using two arrays $V_{EB}$ and $N_{B}$, as in (4) and (5):

(4) $$\begin{array}{c} V_{EB}=\left\{\begin{array}{c} V_{EB1}\\ V_{EB2}\\ V_{EB3}\\ .\\ .\\ V_{EBn} \end{array}\right\} \end{array}$$

(5) $$\begin{array}{c} N_{B}=\left\{\begin{array}{c} N_{B1}\\ N_{B2}\\ N_{B3}\\ .\\ .\\ N_{Bn} \end{array}\right\} \end{array}$$

where *n* is the number of elements in the particular class. We define a vector that contains the length for each vector in the array, as mentioned in (6) and (7):

(6) $$\begin{array}{c} L_{V_{EB}}=\left\{\begin{array}{c} Length(V_{EB1})\\ Length(V_{EB2})\\ Length(V_{EB3})\\ .\\ .\\ Length(V_{EBn}) \end{array}\right\} \end{array}$$

(7) $$\begin{array}{c} L_{N_{B}}=\left\{\begin{array}{c} Length(N_{B1})\\ Length(N_{B2})\\ Length(N_{B3})\\ .\\ .\\ Length(N_{Bn}) \end{array}\right\} \end{array}$$

Subsequently, we compute the minimum among all of the vectors in $L_{V_{EB}}$ and $L_{N_{B}}$, and alter the size of the vectors in the array by Algorithm 1, producing the new signals. Algorithm 1 is explained in a general form that can be considered for any vectors that belong to the same class in the dataset.

$$min_{L_{class}}=minimum\left(L_{class}\right)$$

**Algorithm 1** Changing the size of vectorsInitialize **M** as new matrix **for** i = 1 to n **do**      l←lengthofclass      **if**
$l=min_{L_{class}}$
**then**          Append class to M      **else**          rational fraction approximation of $min_{L_{class}}$ and *l*      **end if**
**end for**

$R_{S}\leftarrow Resampled\;classes$
Append $R_{S}$ to *M*


### 4.3. SynSigGAN: Generative Adversarial Networks

In this section, we utilize the preprocessed signals to generate synthetic biomedical signals using generative adversarial networks. GAN consists of two networks, namely generator (G) and discriminator (D), which compete against each other in a zero-sum game. The generator is provided with random input noise variable $p_{z}\left(z\right)$ from which the generator captures the data distribution $p_{d}$ over the data *x*. The sample that is generated by the generator and original input ground-truth data is fed to the discriminator. The task of the discriminator is to correctly evaluate the generator’s sample with respect to real data. Generator loss is the penalty for the generator if it fails to fool the discriminator. A point comes when the generator outputs excellent samples that are almost similar to the original data and the discriminator gets worse at distinguishing real from generated data. Therefore, the generator aims to minimize log(1−D(G(z)) and the value function $F_{v}\left(D,G\right)$ in the minmax game is defined as in (8). We have proposed a novel GAN architecture that uses a bidirectional grid long short-term memory (BiGridLSTM) for the generator network and a convolutional neural network for the discriminator network.

(8) $$\min_{G}\;\max_{D}\;F_{v}\left(D,G\right)=E_{x∼p_{d}\left(x\right)}\left[logD\left(x\right)\right]+E_{z∼P_{z}\left(z\right)}\left[log\left(1-D\left(G\left(z\right)\right)\right)\right]$$

**Figure 4:**
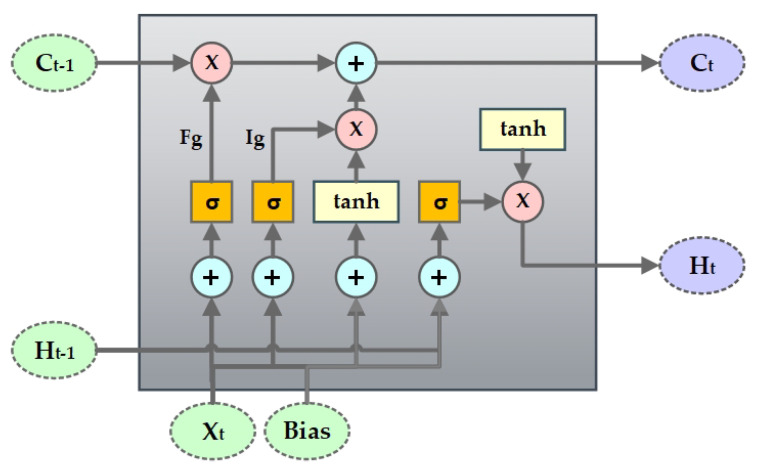
Long Short-Term Memory (LSTM) Layout.

Hochreiter & Schmidhuber [31] introduced long short-term memory (LSTM), which could avoid the long-term dependency problem that is faced by recurrent neural networks (RNN) and remember information over a longer period of time. The LSTM has four interacting layers of neural network with a chain like structure of repeating modules as RNN. Cell state is the core component of the LSTM network that helps information to flow through the entire network. LSTM can, at any point, add or remove information from the cell state. The sigmoid or the forget gate $F_{g}$ layer makes the decision of which information is needed to be eliminated from the cell state, as shown in Figure 4. It takes in $H_{t-1}$ and $X_{t}$ and outputs 0 (denoting information removal) or 1 (denoting keep information) for each cell state $C_{t-1}$. The next part is to determine what information we want to store in the cell state. For this, the sigmoid or the input gate $I_{g}$ layer first decides which value we need to update and then the tanh layer creates a vector $C_{t}$ with new values to be included in the cell state. Subsequently, they are combined in order to obtain a new state and update the existing state. Like any nodes in neural network, the gates in the LSTM network use weights to traverse or filter information and they can be adjusted based on the learning process.

The LSTM network at a time step *t* can be formulated as in (9) [32].

(9) $$\begin{array}{c} I_{t}=\sigma \left(A_{I}X_{t}+B_{I}H_{t-1}+D_{I}C_{t-1}+g_{I}\right)\\ F_{t}=\sigma \left(A_{F}X_{t}+B_{F}H_{t-1}+D_{F}C_{t-1}+g_{F}\right)\\ {\hat{C}}_{t}=\tanh\left(A_{C}X_{t}+B_{C}H_{t-1}+g_{C}\right)\\ C_{t}=F_{t}\circ C_{t-1}+I_{t}\circ {\hat{C}}_{t}\\ O_{t}=\sigma \left(A_{O}X_{t}+B_{O}H_{t-1}+D_{O}C_{t}+g_{O}\right)\\ H_{t}=A_{proj}^{\prime }\left(O_{t}\circ \tanh\left(C_{t}\right)\right) \end{array}$$

where, *A*, *B*, and *D* represent the weight matrix and *g* represents the bias vector. σ is the sigmoid activation function, ∘ represents the element-wise product, and $A_{proj}^{\prime }$ denotes the projection matrix.

Grid Long Short-Term Memory (GridLSTM) was first introduced in 2015 [33], which is an improved version of LSTM that could represent the LSTM blocks into a multidimensional grid. The advantage of GridLSTM is that it can deploy cells along any dimension of the network. For our proposed work, we have considered GridLSTM along two dimensions, i.e., temporal as one of the dimension and depth as the second. Along every dimension, the GridLSTM is linearly related to the gates among all of the adjacent cells, eliminating the vanishing gradient problem along each dimension. The calculation of the time and depth LSTM block in our proposed work can be defined as in (10) and (11).

Time-LSTM block in Grid-LSTM:

(10) $$\begin{array}{c} I_{t,l}^{Tm}=\sigma \left(A_{I,l}^{dep}H_{t,l-1}^{dep}+B_{I,l}^{Tm}H_{t-1,l}^{Tm}+D_{I,l}^{Tm}C_{t-1,l}^{Tm}+g_{I,l}^{Tm}\right)\\ F_{t,l}^{Tm}=\sigma \left(A_{F,l}^{dep}H_{t,l-1}^{dep}+B_{F,l}^{Tm}H_{t-1,l}^{Tm}+D_{F,l}^{Tm}C_{t-1,l}^{Tm}+g_{F,l}^{Tm}\right)\\ {\hat{C}}_{t,l}^{Tm}=\tanh\left(A_{C,l}^{dep}H_{t,l-1}^{dep}+B_{C,l}^{Tm}H_{t-1,l}^{Tm}+g_{C,l}^{Tm}\right)\\ C_{t,l}^{Tm}=F_{t,l}^{Tm}\circ C_{t-1,l}^{Tm}+I_{t,l}^{Tm}\circ {\hat{C}}_{t,l}^{Tm}\\ O_{t,l}^{Tm}=\sigma \left(A_{O,l}^{dep}H_{t,l-1}^{dep}+B_{O,l}^{Tm}H_{t-1,l}^{Tm}+D_{O,l}^{Tm}C_{t,l}^{Tm}+g_{O}^{Tm}\right)\\ H_{t,l}^{Tm}=A_{proj,l}^{{}^{\prime }Tm}\left(O_{t,l}^{Tm}\circ \tanh\left(C_{t,l}^{T}\right)\right) \end{array}$$

**Figure 5:**
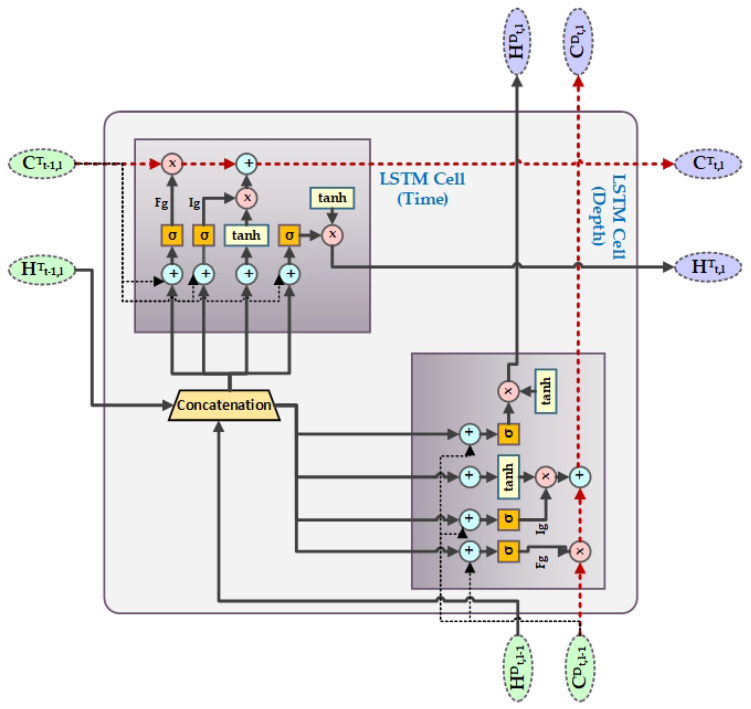
Grid Long Short-Term Memory (GridLSTM) Layout.

Depth-LSTM block in GridLSTM:

(11) $$\begin{array}{c} I_{t,l}^{dep}=\sigma \left(A_{I,l}^{dep}H_{t,l-1}^{dep}+B_{I,l}^{Tm}H_{t-1,l}^{Tm}+D_{I,l}^{dep}C_{t,l-1}^{dep}+g_{I,l}^{dep}\right)\\ F_{t,l}^{dep}=\sigma \left(A_{F,l}^{dep}H_{t,l-1}^{dep}+B_{F,l}^{Tm}H_{t-1,l}^{Tm}+D_{F,l}^{dep}C_{t,l-1}^{dep}+g_{F,l}^{dep}\right)\\ {\hat{C}}_{t,l}^{dep}=\tanh\left(A_{C,l}^{dep}H_{t,l-1}^{dep}+B_{C,l}^{Tm}H_{t-1,l}^{Tm}+g_{C,l}^{dep}\right)\\ C_{t,l}^{dep}=F_{t,l}^{deo}\circ C_{t,l-1}^{dep}+I_{t,l}^{dep}\circ {\hat{C}}_{t,l}^{dep}\\ O_{t,l}^{dep}=\sigma \left(A_{O,l}^{dep}H_{t,l-1}^{dep}+B_{O,l}^{Tm}H_{t-1,l}^{Tm}+D_{O,l}^{dep}C_{t,l}^{dep}+g_{O,l}^{dep}\right)\\ H_{t,l}^{dep}=A_{proj,l}^{{}^{\prime }dep}\left(O_{t,l}^{dep}\circ \tanh\left(C_{t,l}^{dep}\right)\right) \end{array}$$

where dep represents depth dimension and Tm represents time dimension. Whereas, *t* represents time and *l* denotes the number of layers. For example, $H_{t,2}^{dep}$ represents the cell state of depth dimension in the depth-LSTM block of the second layer at time *t*. Initially, we set the value of $C_{t,0}^{dep}$ to zero. In our proposed word, *A* in GridLSTM is the weight between the gating unit and output of the upper layer grid of the depth dimension, whereas *B* is the weight between the gating unit and output of the previous instant of the time dimension. *D* can be considered as the weight of the cell states along every dimension of contiguous grid in the LSTM blocks. Figure 5 shows the structure of GridLSTM.

**Figure 6:**
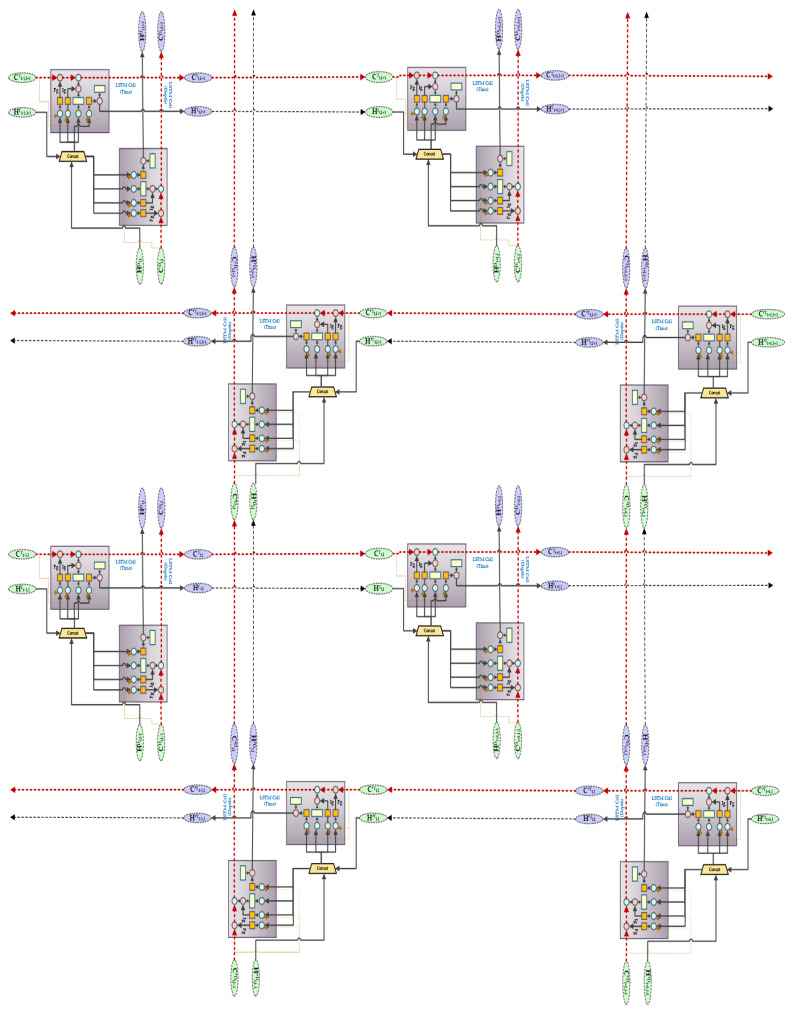
Bidirectional Grid Lon Short-Term Memory (BiGridLSTM) Architecture.

We have used a bidirectional grid LSTM for our generator network in the GAN architecture that integrates the GridLSTM with a bidirectional architecture. BiGridLSTM is a combination of a double GridLSTM in opposite directions, each containing a time and depth LSTM block. The BiGridLSTM has the advantage of diminishing the gradient phenomenon from two dimensions and obtaining the context information at the same time frame. It has also proved to produce excellent outcomes in time sequence problems. The output of the hidden layer of a GridLSTM in forward direction has the current instant as an input and previous moment as the output. In the proposed architecture the output of the hidden layer 1 at the time *t* towards the direction of the depth and in time dimension are impacted by the output $H_{t+1,l}^{Tm}$ at time t+1 of the hidden layer 1 in the time dimension and input $X_{t}^{dep}$ in the depth direction at time *t*; or by the output of $H_{t,l-1}^{dep}$ at time t of the hidden layer l−1 in the reverse GridLSTM. The final output of the BiGridLSTM is the synthetic biomedical signals by combining and connecting the outputs of the forward and reverse GridLSTM. BiGridLSTM overcomes the problem of capturing context information by scanning the input sequences together and by eliminating vanishing gradient problem in the vertical dimension. The time and depth LSTM in the reverse GridLSTM can be defined as in (12) in simple form [32].

(12) $$\begin{array}{c} \left(H_{t,l}^{{}^{\prime }Tm},C_{t,l}^{{}^{\prime }Tm}\right)=Time\_LSTM(H_{t,l-1}^{{}^{\prime }dep},H_{t+1,l}^{{}^{\prime }Tm},C_{t+1,l}^{{}^{\prime }Tm},{⊙}^{{}^{\prime }Tm}\\ \left(H_{t,l}^{{}^{\prime }dep},C_{t,l}^{{}^{\prime }dep}\right)=Depth\_LSTM(H_{t,l-1}^{{}^{\prime }dep},H_{t+1,l}^{{}^{\prime }Tm},C_{t,l-1}^{{}^{\prime }Tm},{⊙}^{{}^{\prime }dep} \end{array}$$

where ${}^{\prime }$ represents the reverse GridLSTM block and ⊙ implies all of the parameters in the inverse LSTM block. The combined output of the hidden layer *l* at a given time *t* is defined as in Equation (13). Figure 6 shows the BiGridLSTM architecture that is used for generating synthetic biomedical signals.

(13) $$\begin{array}{c} Output_{t,l}=GridLSTM\left(H_{t,l}^{dep},H_{t,l}^{{}^{\prime }dep}\right) \end{array}$$

**Figure 7:**
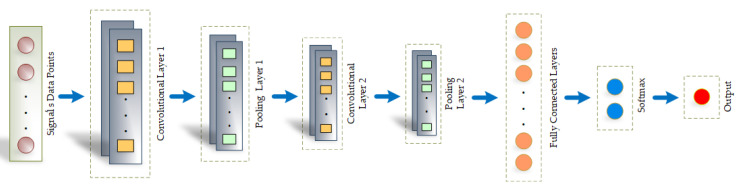
Discriminator Architecture with Convolutional Neural Network.

The sample of synthetic signals that are generated from the generator is then passed to the discriminator along with the original signal. The task of the discriminator is to evaluate and distinguish the real data from the data created by the generator. Hence, as the training proceeds, the generator tries to fool the discriminator, which consequently improves the quality of the synthetic signals generated. We have built our discriminator architecture with convolutional neural network, as shown in Figure 7. We have used a 1-D convolutional network that takes, as input, *T* data points, each represented by n-dimensional vector. The filter size has been set to H*1 and stride to P∗1 (5∗1 and 3∗1). Accordingly, the size of the output of the first convolutional layer can be defined as [(No. ofFilters)∗[(T−H)/P+1]∗1]. Subsequently, there are the max pooling layers and the second convolutional and pooling layer. The fully connected layer maps the softmax layer that outputs a one-hot vector. There are two components in the vector that showcase the probability of the input being true or false and, finally, outputs the decision of the discriminator, as we can see from Figure 7.

## 5. Evaluation and Results of the Proposed Approach

The metrics that we have used for evaluating the quality of the generated synthetic signals are Root Mean Square Error (RMSE), Percent Root Mean Square Difference (PRD), Mean Absolute Error (MAE), and Fréchet Distance (FD). We have also calculated the Pearson’s Correlation Coefficient (PCC) between the original and generated synthetic signal for statistical analysis.

### 5.1. Root Mean Square Error

We use the root mean square error to measure the stability between the original signal (O) and the generated synthetic signal (S) by SynSigGAN. It can be formulated, as shown in (14):

(14) $$\begin{array}{c} RMSE=\sqrt{N\sum_{i=1}^{N}{\left(O_{i}-S_{i}\right)}^{2}} \end{array}$$

### 5.2. Percent Root Mean Square Difference

The percent root mean square has been used to calculate the distortion between two signals, as shown in (15):

(15) $$\begin{array}{c} PRD=\sqrt{100\frac{\sum_{i=1}^{N}{\left(O_{i}-S_{i}\right)}^{2}}{\sum_{i=1}^{N}{\left(O_{i}\right)}^{2}}} \end{array}$$

### 5.3. Mean Absolute Error

The mean absolute error calculates the average of the absolute differences between the original and synthetic signals while using Equation (16):

(16) $$\begin{array}{c} MAE=\frac{1}{N}\sum_{i=1}^{N}\left|O_{i}-S_{i}\right| \end{array}$$

### 5.4. Fréchet Distance

We have measured the Fréchet Distance to find the similarity between the ordering and location of points along the curve. If $O_{R}=a_{1},a_{2},a_{3},\ldots ,a_{R}$ is the order of points along the segmented original curves and $O_{S}=b_{1},b_{2},b_{3},\ldots ,b_{S}$ is the order of points along the segmented synthetic curve, then the length ∥l∥ of the sequence can be measured, as in (17):

(17) $$\begin{array}{c} ∥l∥=\max_{i=1,\ldots ,n}l\left(a_{u_{i}},b_{v_{i}}\right) \end{array}$$

where *l* is the euclidean distance and $a_{u_{i}}$ and $b_{v_{i}}$ are the sequence of the order of points. Accordingly, the Fréchet Distance can be calculated as (18) [9]:

(18) $$\begin{array}{c} FD(R,S)=\min∥l∥ \end{array}$$

### 5.5. Pearson’s Correlation Coefficient

The relationship between two variables is measured by the correlation coefficient, which ranges between −1 and +1. Whereas, 0 indicates there is no relationship. A positive value refers to a direct relation and a negative value refers to inverse relation. The Pearson’s correlation coefficient in our proposed methodology was measured as (19) [34]

(19) $$\begin{array}{c} PCC=\frac{\sum_{i=1}^{N}\left(O_{i}-O\right)\left(S_{i}-S\right)}{\sqrt{\left[\sum_{i=1}^{N}\left(O_{i}-\bar{O}\right)\right]\left[\sum_{i=1}^{N}\left(S_{i}-\bar{S}\right)\right]}} \end{array}$$

The correlation values can be transcribed, as in Table 2.

Table 3 and Table 4 show the evaluation results of the signals and different models as compared to our proposed approach.

### 5.6. Results

**Figure 8:**
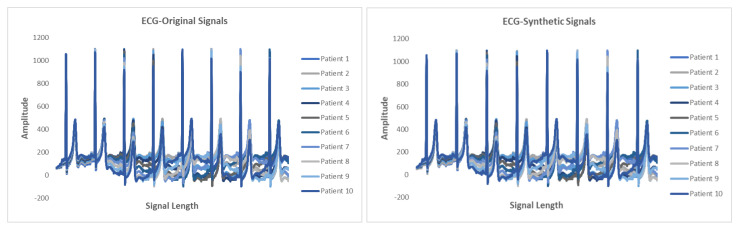
Comparison between original and synthetic ECG signals.

**Figure 9:**
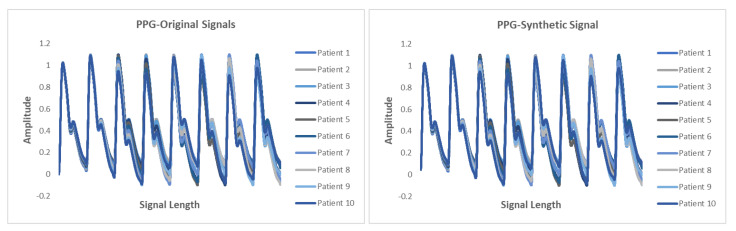
Comparison between original and synthetic PPG signals.

**Figure 10:**
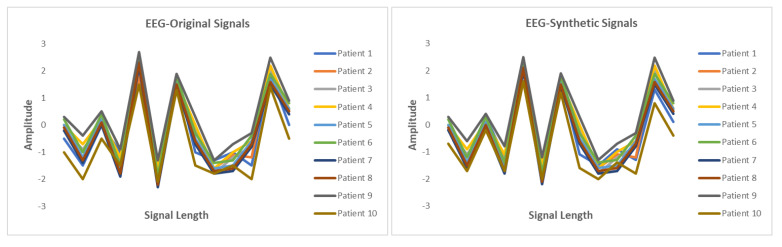
Comparison between original and synthetic EEG signals.

**Figure 11:**
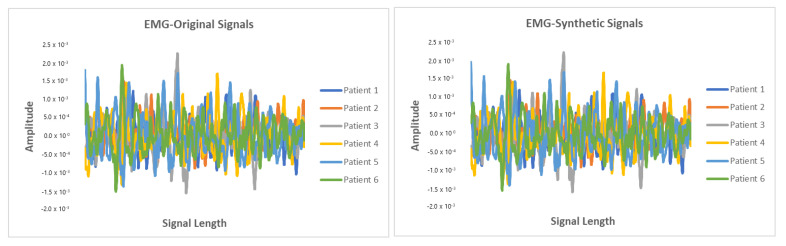
Comparison between original and synthetic EMG signals.

**Figure 12:**
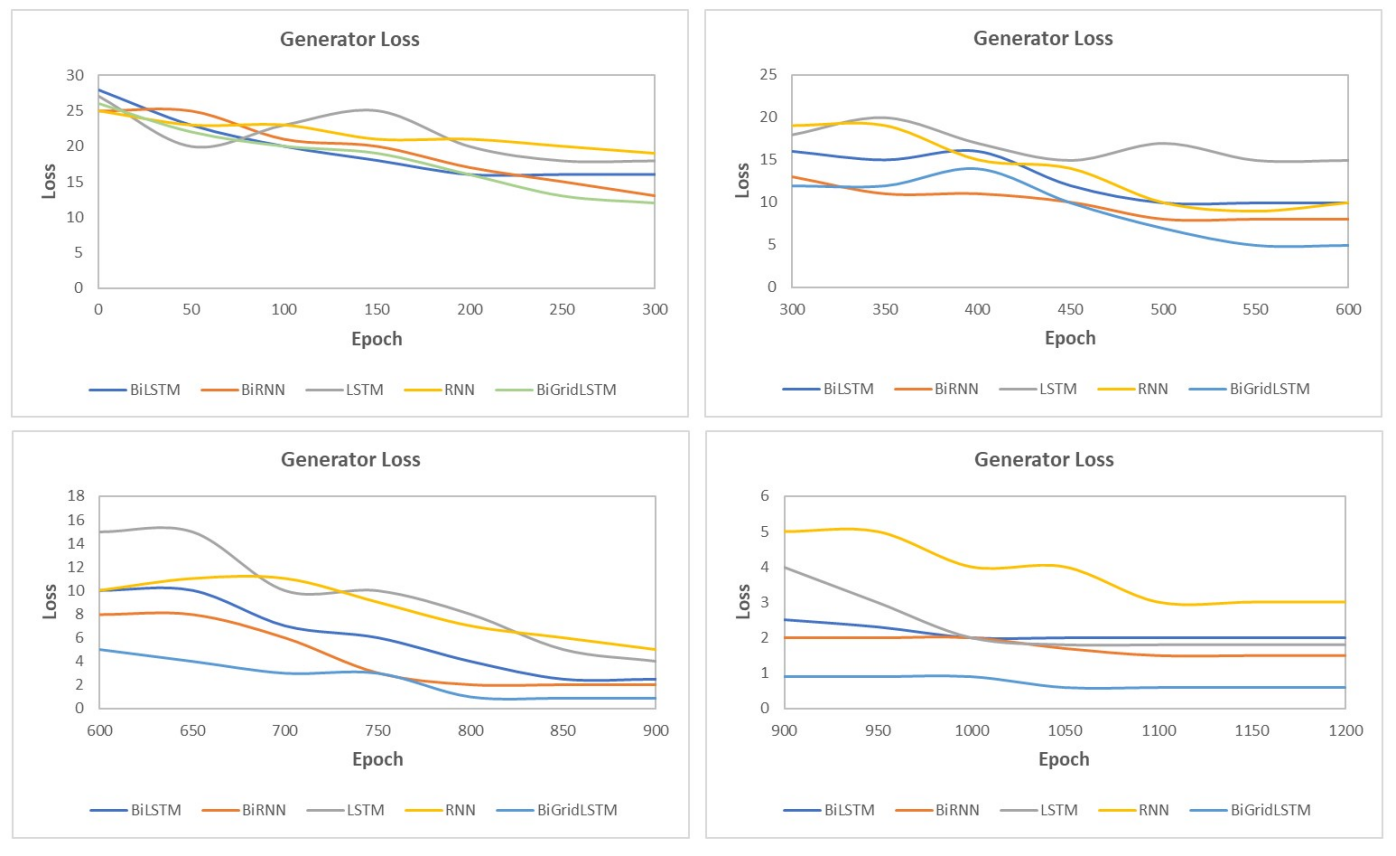
Loss for different generators with CNN as the discriminator in the generative adversarial networks (GAN) architecture.

**Figure 13:**
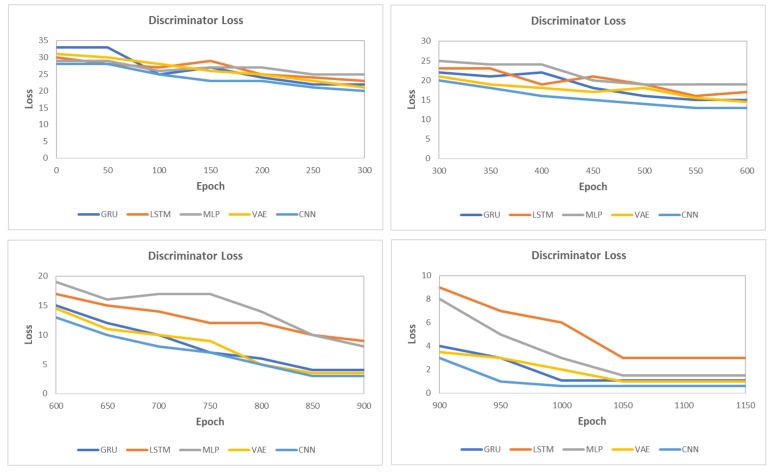
Loss for different discriminators with BiGridLSTM as the generator in the GAN architecture.

This section presents the synthetic signal generation as compared to the original signals for four kinds of biomedical signals. We have shown the result of 10 patients for ECG, PPG, and EEG recordings and six patient for EMG recordings, where every patient has a different period of recording and a different number of signals. Our proposed methodology has been able to obtain a similar quantity of synthetic data as compared to original signals. We have applied our proposed approach on open and closed-eye EEG data and 17 types of ECG signals where there are same number of signals for every class and they also have the same length. Figure 8, Figure 9, Figure 10 and Figure 11 show the comparison of the original and synthetic data generation using our proposed approach. Table 5, Table 6 and Table 7 show the count of original signals and the count for the total number of synthetic signals generated for specific signal types. In this section, we have also presented different generator loss keeping the discriminator network in the GAN architecture as CNN and discriminator loss keeping the generator in the GAN architecture as BiGridLSTM. Figure 12 and Figure 13 depict the generated loss. This evaluation depicts that the combination of BiGridLSTM-CNN in the GAN architecture produces the best result. For evaluation, the synthetic signals that were delivered by our proposed model were used by other models for classification and they resulted in classifying each signal with significantly high accuracy.

## 6. Conclusions

We proposed a generative adversarial networks model, named as SynSigGAN, which was successfully able to generate synthetic biomedical signals with a high correlation coefficient. This paper presented a preprocessing stage that could eliminate noise and refine any kinds of biomedical signals for further processing and to help generate high quality synthetic signals. The model segmented each signal according to the annotations and trained the adversarial networks consisting of the combination of bidirectional grid long short-term memory and convolutional neural network in order to generate realistic synthetic biomedical signals.

The generation of the synthetic signals eliminates privacy concerns and the problem of accessibility of medical data among the researchers. We have used the MIT-BIH arrhythmia database for ECG signals, Sienna scalp database for EEG signals, Sleep EDF database for EMG signals, and BIDMC PPG and respiration dataset for PPG signals. We have compared the correlation between original signals and the synthetic signals generated by our proposed approach. The synthetic signals generated by our proposed approach are highly correlated to the original signals and of remarkable quality, as shown in the results section. Our model could generate signals of various lengths and characteristics. We evaluated our model based on MAE, RMSE, PRD, and FD scores, which were compared with existing models, and the outcome shows that our model performs significantly better than existing models.

## Figures and Tables

**Table 1:** Performance Comparison for Denoising Techniques.

Methodology	SNR	RMSE	PRD
Low Pass Filtering	16.34	6.01	17.89
Short-Time Fourier Transform	17.21	6.56	18.90
Fast Fourier Transform	15.99	7.10	17.34
Wigner-Ville Distribution	14.60	7.82	16.51
Least Mean Squares	16.91	6.11	16.01
Frequency-Domain Adaptive Filtering	17.83	8.25	17.88
Wavelet Transformation	19.82	4.56	14.34

**Table 2:** Representation of Correlation Values.

Correlation Values	Representation
0 to 0.3 or 0 to −0.3	Negligibly correlated
0.3 to 0.5 or −0.3 to −0.5	Low correlation
0.5 to 0.7 or −0.5 to −0.7	Moderately correlated
0.7 to 0.9 or −0.7 to −0.9	Highly correlated
0.9 to 1 or −0.9 to 1	Extensively correlated

**Table 3:** Statistical Measure for Biomedical Signals.

Signal	Mean Correlation Coeficient	MAE	RMSE	PRD	FD
ECG	0.9991	0.218	0.126	6.343	0.936
EEG	0.997	0.0475	0.0314	5.985	0.982
EMG	0.9125	0.0538	0.0529	2.971	0.921
PPG	0.9793	0.0635	0.0596	5.167	0.783

**Table 4:** Evaluation Metric Comparison with Existing Models.

Model	MAE	RMSE	PRD	FD
BiLSTM-GRU	0.59	0.57	74.46	0.95
BiLSTM-CNN GAN	0.5	0.51	68.83	0.89
RNN-AE GAN	0.79	0.76	119.34	0.97
BiRNN	0.6	0.62	89.97	0.96
LSTM-AE	0.77	0.79	148.67	0.99
BiLSTM-MLP	0.75	0.78	146.35	0.99
LSTM-VAE GAN	0.71	0.72	144.74	0.98
RNN-VAE GAN	0.71	0.72	145.22	0.98
BiGridLSTM-CNN	0.36	0.25	66.21	0.79

**Table 5:** Details of Synthetic Photoplethysmography (PPG) Signals Generated.

Patient	Length of Signal	Original Signal Data	Synthetic Signal Data Generated
Patient 1	125	20	20
Patient 2	63	500	500
Patient 3	71	17	17
Patient 4	141	92	92
Patient 5	139	72	72
Patient 6	40	11	11
Patient 7	93	101	101
Patient 8	68	162	162
Patient 9	117	14	14
Patient 10	62	700	700

**Table 6:** Details of Synthetic Electroencephalogram (EEG) Signals Generated.

	Length of Signal	Original Signal Data	Synthetic Signal Data Generated
**Open-Eye**	19	8100	8100
**Closed-Eye**	18	6173	6173

**Table 7:** Details of Synthetic Electromyography (EMG) Signals Generated.

Patient	Length of Signal	Original Signal Data	Synthetic Signal Data Generated
Patient 1	78	70	70
Patient 2	16	11	11
Patient 3	43	191	191
Patient 4	21	55	55
Patient 5	90	41	41
Patient 6	112	17	17
Patient 7	143	54	54
Patient 8	66	71	71
Patient 9	156	15	15
Patient 10	191	19	19
